# A Novel Biochemical Study of Anti-Dermal Fibroblast Replicative Senescence Potential of *Panax Notoginseng* Oligosaccharides

**DOI:** 10.3389/fphar.2021.690538

**Published:** 2021-06-30

**Authors:** Lu Zhai, Xiaohao Xu, Jiangzeng Liu, Chenxu Jing, Xinzhao Yang, Daqing Zhao, Rui Jiang, Li-Wei Sun

**Affiliations:** ^1^ Research Center of Traditional Chinese Medicine, the Affiliated Hospital to Changchun University of Chinese Medicine, Changchun, China; ^2^ Key Laboratory of Active Substances and Biological Mechanisms of Ginseng Efficacy, Ministry of Education, Changchun University of Chinese Medicine, Changchun, China

**Keywords:** panax notoginseng oligosaccharides, replicative senescence dermal fibroblast, cell cycle, cell migration, COL-I

## Abstract

Dermal fibroblast replicative senescence that often occurs in aging skin is characterized by loss of cell proliferative capacity, cell cycle arrest, decreased cell elongation, and decreased synthesis of dermal extracellular matrix (ECM) components. Although *Panax notoginseng* is known for its effectiveness in alleviating many age-related degenerative diseases, few studies have evaluated *P. notoginseng* components for efficacy or mechanisms of action in delaying cell replicative senescence. In this study, *P. notoginseng* oligosaccharides (PNO) were isolated using a stepwise purification procedure involving water extraction and alcohol precipitation followed by DEAE Sepharose Fast Flow column chromatography, preparative high performance liquid chromatography, and size-exclusion chromatography. Monosaccharides detected in PNO constituents included mannose, galactose, and sorbitose in relative molar proportions of 14.2:12.3:1, respectively, aligning with PNO absorption spectrum results resembling typical known spectra for sugars. *In vitro*, PNO treatment of replicative senescent NIH-3T3 fibroblasts significantly promoted cell vitality, inhibited SA-β-galactosidase (SA-β-Gal) activity, and reduced p16 and p21 protein-level expression. Moreover, PNO treatment of senescent fibroblasts led to a lower proportion of G1 phase cells and higher proportion of S phase cells, while also inducing aging NIH-3T3 cells to migrate and synthesize collagen-I (CoL-I). Mechanistically, PNO treatment up-regulated expression of proliferating cell nuclear antigen (PCNA), cyclin E, cyclin D1, and cyclin-dependent kinase 4 (CDK4) proteins and promoted phosphorylation of MEK, p38, and ERK1/2 to trigger cell cycle progression. Additionally, PNO treatment also up-regulated protein-level expression of TGF-β1 and levels of p-Smad2/3, p-FAK, and p-Pax to trigger CoL-I synthesis and cell migration. Taken together, these findings demonstrate that oligosaccharides purified from *P. notoginseng* could reverse fibroblast replicative senescence by promoting fibroblast cell proliferation, migration, and CoL-I production.

## Introduction

As people get older, skin undergoes endogenous aging that mainly manifests itself as thinning of skin leading to atrophic appearance, reduced elasticity, fine wrinkles, loss of underlying fat, dryness, and increased sensitivity (Gu et al., 2020). Major pathological changes associated with aging skin include epidermal layer thinning accompanied by epidermal rete ridge loss, dermal papillae attenuation, extracellular matrix atrophy, and decreases in numbers of fibroblasts, collagen fibers, and elastic fibers (Russell-Goldman and Murphy, 2020). As key cellular components of skin, dermal fibroblasts maintain dermal extracellular matrix (ECM) homeostasis by degrading depleted dermal components and synthesizing new components to rebuild and renew the ECM and maintain skin structure (Sadowska-Bartosz and Bartosz, 2020). In aged skin, prominent age-related dermal fibroblast characteristics include reduced cell size, decreased cell elongation, and decreased cell ability to synthesize ECM (Kanaki et al., 2016). These changes appear to be related to a fibroblast-aging phenomenon known as replicative senescence. Cells underdoing replicative senescence cease to differentiate in culture after a certain number of replicative cycles, as demonstrated in studies of human and murine fibroblasts and other epidermally derived cells (Mohamad Kamal et al., 2020). Replicative senescent fibroblasts tend to exhibit a stagnated or underactive cell replication cycle associated with increased SA-β-galactosidase (SA-β-Gal) activity and increased expression of commonly observed senescence-related markers p16 and p21 (Sadowska-Bartosz and Bartosz, 2020). Moreover, replicative senescence has been linked to telomere shortening and imbalanced oxidative stress responses (Mohamad Kamal et al., 2020; Sadowska-Bartosz and Bartosz, 2020).

Fibroblast entry into a senescent state begins with cell cycle arrest in the G1 cell phase (Sadowska-Bartosz and Bartosz, 2020). The inability of arrested cells to progress from G1 to S phase results from reduced expression of cell proliferation antigen (PCNA) that is associated with decreased expression and activity of cell cycle-dependent kinases (CDK) and cyclins (Chen et al., 2018). Meanwhile, levels of other functional cellular biological activities, such as cell migration, are also reduced in senescent cells (Phillip et al., 2017), including focal adhesion kinase (FAK)/paxillin (Pax) signaling pathway activity, which plays a major role in promoting cell migration (Yu et al., 2009). Additionally, production of type I collagen (CoL-I), the primary dermal matrix component regulated mainly through TGF-β1/Smad signaling pathways (Zhang et al., 2012), is also reduced during cell entry into replicative senescence, leading to reduced skin tensile strength, loss of elasticity, and formation of wrinkles (Brun et al., 2016).

*P. notoginseng* (Burkill) F. H. Chen, commonly known as “Sanqi” in China, belongs to the family Araliaceae (Liu et al., 2020). Its medicinal properties have been attributed to active components such as saponins, polysaccharides, volatile oils, flavonols, glycosides, amino acids, etc. (Choi et al., 2010; Liu et al., 2021). Studies have shown that treatment of skin with *P. notoginseng* can accelerate wound healing in diabetic patients *via* multiple processes that include promotion of fibroblast proliferation (Zhang et al., 2016). Additional research has shown that *P. notoginseng* may also exert anti-cancer and anti-aging effects, the latter of which includes cell lifespan prolongation and prevention of vascular and brain cell aging (Zhao et al., 2017). Based on the abovementioned research, we speculate that *P. notoginseng* treatment may delay natural skin aging by promoting fibroblast proliferative activity. Bouaziz et al. reported that oligosaccharides from various sources exhibited anti-photoaging healing effects when used to treat dermal wounds (Bouaziz et al., 2014; Kong et al., 2018; Suh et al., 2020). Therefore, in this study we processed *P. notoginseng* using a variety of preparative techniques and isolated oligosaccharides, an efficacious mixture that prevented fibroblast replicative senescence. After characterizing components responsible for the anti-aging activity, we explored underlying mechanisms for this effect by developing and implementing an experimental methodology to evaluate anti-aging activities of herbal remedies, including *P. notoginseng*, and their constituents.

## Material and Methods

### Materials

Fibroblasts (murine cell line, NIH-3T3 cells) obtained from the Cell Resource Center of the Shanghai Institute for Biological Sciences (Shanghai, China) were used in all experiments. Cells were cultured in DMEM and newborn bovine serum (NBS) (Invitrogen, Carlsbad, CA, United States). Other reagents (DMSO, MTT, penicillin, streptomycin, Triton X-100) were obtained commercially (Sigma-Aldrich, St. Louis, MO, United States). Mouse SA-β-galactosidase, p16, and p21 ELISA Kits were purchased from the Beyotime Institute of Biotechnology (Shanghai, China). Mouse monoclonal antibodies against p16, p21, CoL-I, PCNA, cyclin D1, cyclin E, CDK4, FAK/p-FAK, Pax/p-Pax, MEK/p-MEK, ERK/p-ERK, p38/p-p38, TGF-β1, Smad 2/3/p-Smad 2/3, GAPDH, and β-actin were purchased from Abcam. For western blotting, luminol reagent was purchased from Santa Cruz Biotechnology (Dallas, TX, United States). Sheep anti-rat secondary antibody and sheep anti-rabbit secondary antibody were purchased from Beijing Biosynthesis Biotechnology Co., LTD. (Beijing, China). All other reagents were of analytical grade and were produced in China.

### Preparation of *P. notoginseng* Oligosaccharides (PNO)

Plant material (*P. notoginseng* roots) was provided by the Panax Notoginseng Research Institute, (Wenshan, Yunan Province, China) in October 2017. The voucher specimen (2213) was deposited at the Research Center of Traditional Chinese Medicine, The Affiliated Hospital to Changchun University of Chinese Medicine, Jilin Province, China. Dried *P. notoginseng* roots (1,000 g) were crushed to form a 100-mesh powder that was extracted two times with 10 L of distilled water at 100°C under reflux for 1 h. The supernatant was consolidated, concentrated, then precipitates were formed after addition of 75% ethanol and incubation overnight (Li et al., 2020).

The next day, after filtering the mixture, the clarified supernatant was retained and ethanol in the supernatant was removed *via* evaporation. The remaining liquid was transferred to a 50-ml DEAE Sepharose Fast Flow chromatography column. Fractions were serially eluted with phosphate buffer (pH = 7.6) followed by elution with 2 M NaCl. Each eluate was collected automatically as 4-ml fractions into tubes. The absorption curve of eluted sugars (determined at 490 nm) was determined using the phenol sulfuric acid method, then the protein absorption curve was determined using absorbance readings at 280 nm. Fractions with major sugar contents were pooled, dialyzed, then lyophilized (Cheng et al., 2020). The pooled sugar-containing fraction was then diluted and separated using a high performance liquid chromatography (HPLC) system equipped with a Click Xion column (5 μm, 100 A, 10 × 250 mm). The HPLC column temperature was held at 30°C, flow velocity was maintained at 4.0 ml/min for the mobile phase of acetonitrile/water (20:80, v/v); detection was conducted using an evaporative photodetector (ELSD) (drift tube temperature: 80°C; gas flow rate: 2.5 L/min) (Guo et al., 2014; Zhao H. et al., 2020). The oligosaccharide fraction corresponding to the major chromatographic peak was collected. Oligosaccharides were subsequently purified using gel chromatography with an OHpak SB-802.5 HQ column followed by elution with pure water at a flow rate of 0.5 ml/min and detection using a an ELSD photodetector (Nguyen et al., 2013). The oligosaccharide fraction exhibiting peak activity against dermal fibroblast replicative senescence was collected and lyophilized, the resulting solid was assessed to determine the structural characteristics and activities of its components.

### Gas Chromatography-mass Spectrometry (GC-MS)

The monosaccharide composition of PNO was determined using GC-MS with a slight modification (Cheng et al., 2020). In brief, PNO (50 mg) was added to 4 ml of 2 M trifluoroacetic acid (TFA) and hydrolysis proceeded for 2 h at 121°C. Next, 2 ml of methanol was added to the solid to remove excess TFA then the samples were dried and the remaining solid was dissolved in distilled water and reduced with 20 mg NaBH_4_ for 3 h. After neutralization with glacial acetic acid followed by evaporation to dryness, the residue was acetylated with acetic anhydride (4 ml) at 100°C for 1 h. Next, chloroform was added to extract monosaccharides and repeated for a total of at least five chloroform extractions. Extracted monosaccharides were analyzed via GC-MS using a 6890N/5973i GC/MS System (Agilent Technology, CA, United States) equipped with a DB-1 capillary column (30 m × 0.25 mm × 0.25 μm; Thermo, United States).

### Infrared Spectroscopy (IR)

PNO (10 mg) was mixed with dried potassium bromide (KBr) then the mixture was compressed and placed in an infrared spectrometer (Tensor27 type) and analyzed over a frequency range of 400–4,000 cm^−1^ (Zhao B. et al., 2020).

### Cell Culture and Establishment of the Replicative Senescence Cell Model

NIH-3T3 cells were cultured in DMEM at 37°C with 10% NBS and 100 μg/ml streptomycin in a humidified 5% CO_2_ incubator (Thermo Fisher Scientific, Waltham, MA, United States). Cells in logarithmic growth phase were harvested *via* trypsinization, centrifuged, then adjusted to a cell density of 1 × 10^5^ cells/ml with fresh DMEM containing 10% NBS. Next, cells were dispensed into a petri dish (10-cm diameter) for continuous culture. After 8–10 passages, cells exhibited characteristics of young cells (designated T0, the control group), while cells after 45–50 passages exhibited characteristics associated with a state of replicative senescence (designated T1, the replicative senescence model group) (Yang K.-E. et al., 2020).

### Cell Viability Assay

A modified version of the MTT assay was used to test the effects of PNO on NIH-3T3 cell viability (Jiang et al., 2017). NIH-3T3 cells (4 × 10^4^ cells/well) were plated in a 96-well plate for 24 h then culture medium was discarded and replaced with DMEM containing 0.4% NBS followed by incubation of cells for another 24 h. Cells were incubated with PNO at 15.63 μg/ml, 31.25 μg/ml, and 62.5 μg/ml (10 mg PNO was dissolved in DMEM containing 0.4% NBS to 1 mg/ml to create a concentrated stock solution that was sterilized by filtration through a 0.22-μm filter. The stock was then adjusted to the aforementioned concentrations by addition of sterile DMEM containing 0.4% NBS). Meanwhile, the positive control was incubated with epidermal growth factor (EGF) in the same medium for 48 h. Next, MTT was added to each well (10 μl, 5 mg/ml stock solution) then plates were incubated for 4 h at 37°C. Next, each well received 150 µl of DMSO and plates were shaken for 10 min and absorbance readings (570 nm) were recorded using a microplate reader, with all data collected from triplicate samples.

### ELISA

NIH-3T3 cells (4.0 × 10^4^ cells/well) were placed in a six-well plate and cultured using the aforementioned culture method. Next, cells were collected, washed twice with PBS buffer, and lysed in 1% Triton X-100 during a 1-h incubation at 4°C. The supernatant was collected then the protein concentration was measured using a BCA Protein Assay Kit. Next, ELISA was used to detect effects of PNO treatment on SA-β-Gal activity in replicative senescent NIH-3T3 cells according to the manufacturer’s instructions (Baeeri et al., 2020).

### Annexin V-FITC/propidium Iodide Staining

NIH-3T3 cells were incubated in 12-well plates (3 × 10^5^ cells/ml, 2 ml/well) for various time periods, washed with PBS, and resuspended in 50 µl Annexin V-FITC binding buffer (BD Biosciences) at room temperature (RT) then 3 μl of Annexin V-FITC was added followed by incubation for 15 min. Next, 6 μL of propidium iodide (PI) was added to each well then plates were left at room temperature for an additional 5 min followed by analysis of cells using a flow cytometer (FACS Calibur Flow; BD Biosciences). CI values were calculated using CalcuSyn 2.0 software (Biosoft).

### Cell Cycle Assay

Flow cytometry was used to detect effects of PNO on the cell cycle (Cheng et al., 2021). After NIH-3T3 cells (4 × 10^4^ cells/well) were cultured in a six-well plate using the same culture method as mentioned above, the cells were washed twice with ice-cold PBS then incubated in 70% alcohol at 4°C for 2 h and centrifuged. After centrifugation, cell pellets were collected, suspended in PI, then incubated for 30 min at 37°C followed by determination of fluorescence intensity using flow cytometry (C6; BD Biosciences, San Jose, CA, United States).

### Scratch Test

Scratch tests were performed to assess migration ability of treated cells with a slight modification (Cheng et al., 2021). NIH-3T3 cells (9.0 × 10^4^ cells/well) were placed in a 12-well plate and cultured for 24 h. Then a sterile pipette tip was used to gently scratch the cell layer from top to bottom to form a uniform scratch then scratched layers were washed with PBS buffer to remove wound debris. Scraped cells were treated with PNO or EGF then the cells were cultured with serum-free medium in a CO_2_ incubator at 37°C for 24 and 48 h, respectively. After taking pictures of scratches using a multifunctional imager, cell migration distance was measured using Image-J Analysis software.

### Western Blot Analysis

Protein preparations of cells of each experimental group were obtained by treatment of cells with lysis buffer (20 mM Tris–HCl [pH 7.4], 2 mM EDTA, 20 μM NaCl, 20 μM CHAPS, 0.5 mM PMSF) followed by centrifugation (12,000 rpm, 20 min). Supernatants were collected then a BCA Protein Assay Kit was used to determine protein concentration. Next, proteins in each sample (20 μg total protein) were separated via electrophoresis (12% SDS-PAGE gels) followed by transfer of proteins to PVDF membranes. Next, proteins on membranes were incubated overnight at 4°C with various monoclonal antibodies (anti-CoL-I, anti-p16, anti-p21, anti-PCNA, anti-cyclin D1, anti-cyclin E, anti-CDK4, anti-MEK/p-MEK, anti-p38/p-p38, anti-ERK1/2/p-ERK1/2, anti-FAK/p-FAK, anti-Pax/p-PAK, anti-Smad2/3/p-Smad2/3, TGF-β1, GAPDH, and anti-β-actin antibodies). The next day, membranes were washed then incubated with secondary antibodies followed by detection of proteins using chemiluminescent reagents (Jiang et al., 2020).

### Statistical Analysis

All data are represented as mean ± STD of three biological replicates in this study. Statistical analysis was performed using one-way ANOVA and Tukey’s *post hoc* test for multiple comparisons testing. Statistical evaluation was performed using GraphPad Prism, version 5.0 (GraphPad Software, San Diego, CA, United States). *p* < 0.05 was considered statistically significant.

## Results

### Purification and Composition Identification of PNO

**FIGURE 1:**
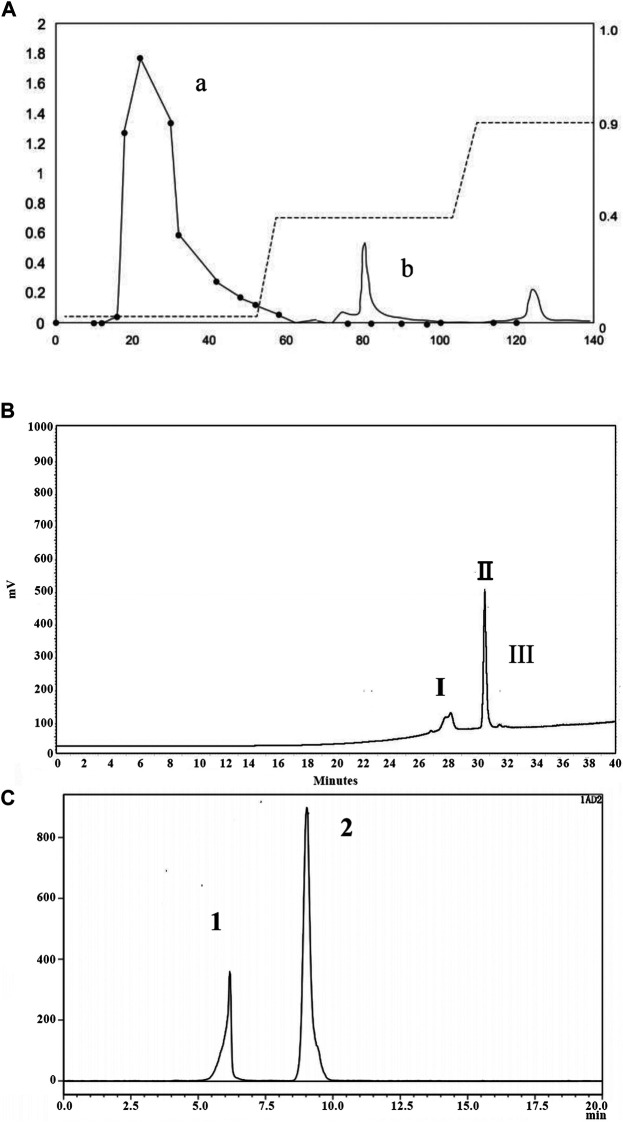
Isolation and purification chromatogram of PNO. **(A)** DEAE column chromatography of PNO. **(a)** Elution curve of sugars obtained using a phenol-sulfuric acid extraction method, with detection conducted *via* absorbance measurements at 490 nm; **(b)** protein elution curve determined from absorbance measurements at 280 nm. **(B)** Separation curve obtained using a Click Xion column *via* HPLC. **(C)** Gel chromatography conducted using an OHpak SB-802.5 HQ column *via* HPLC.

After water extraction of *P. notoginseng* roots followed by alcohol precipitation, extracted components were separated using DEAE Sepharose Fast Flow ion exchange chromatography. As shown in Figure 1A, peak a represented the extract portion with the highest sugar content (91.69%). Subsequently, this portion was further separated using a Click Xion column followed by collection of peak II (Figure 1B). Finally, a gel column was used to separate components according to particle size, resulting in collection of peak I associated with material exhibiting target activity that counteracted dermal fibroblast replicative senescence, *P. notoginseng* oligosaccharide (PNO) (Figure 1C).

**FIGURE 2:**
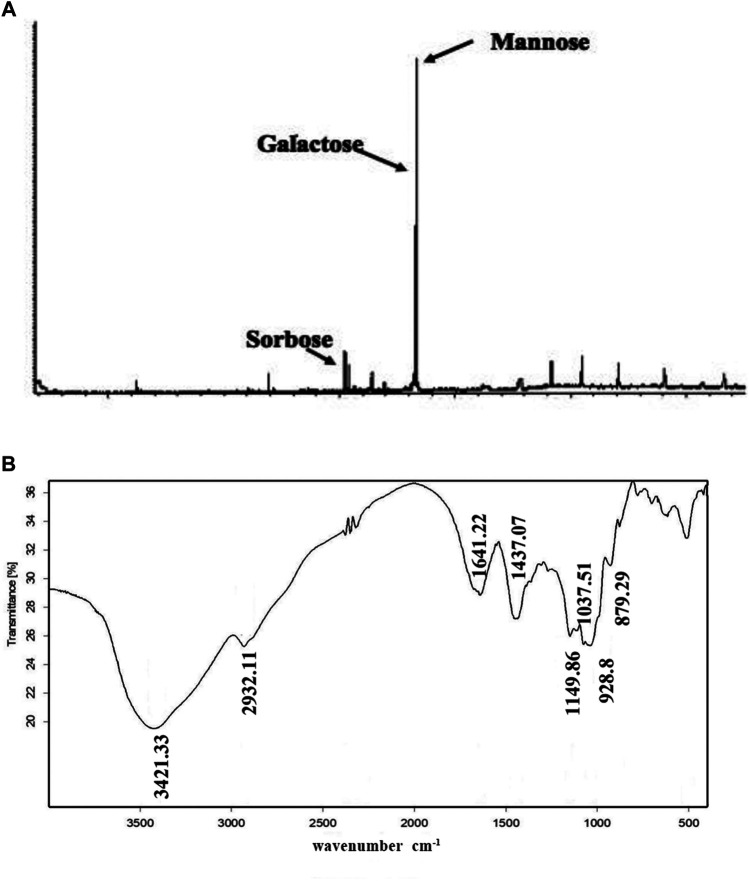
PNO composition and chemical bonds identification. **(A)** PNO composition analysis conducted *via* GC-MS. **(B)** PNO infrared spectroscopic analysis result.

Next, we determined PNO composition and residue identification. As shown in Figure 2A, PNO was shown to be mainly composed of three monosaccharides, mannose, galactose, and sorbose, in a molar ratio of 14.2:12.3:1, respectively. The IR spectrum shown in Figure 2B contained O-H absorption peaks at 3,600–3,200 cm^−1^, C–H absorption peaks at 3,000–2,800 cm^−1^, and pyranose absorption peaks at 1,100–800 cm^−1^. These results suggest that PNO has a typical absorption profile associated with sugars.

### PNO Promotes Cell Vitality in NIH-3T3 Fibroblasts Exhibiting Replicative Senescence

**FIGURE 3:**
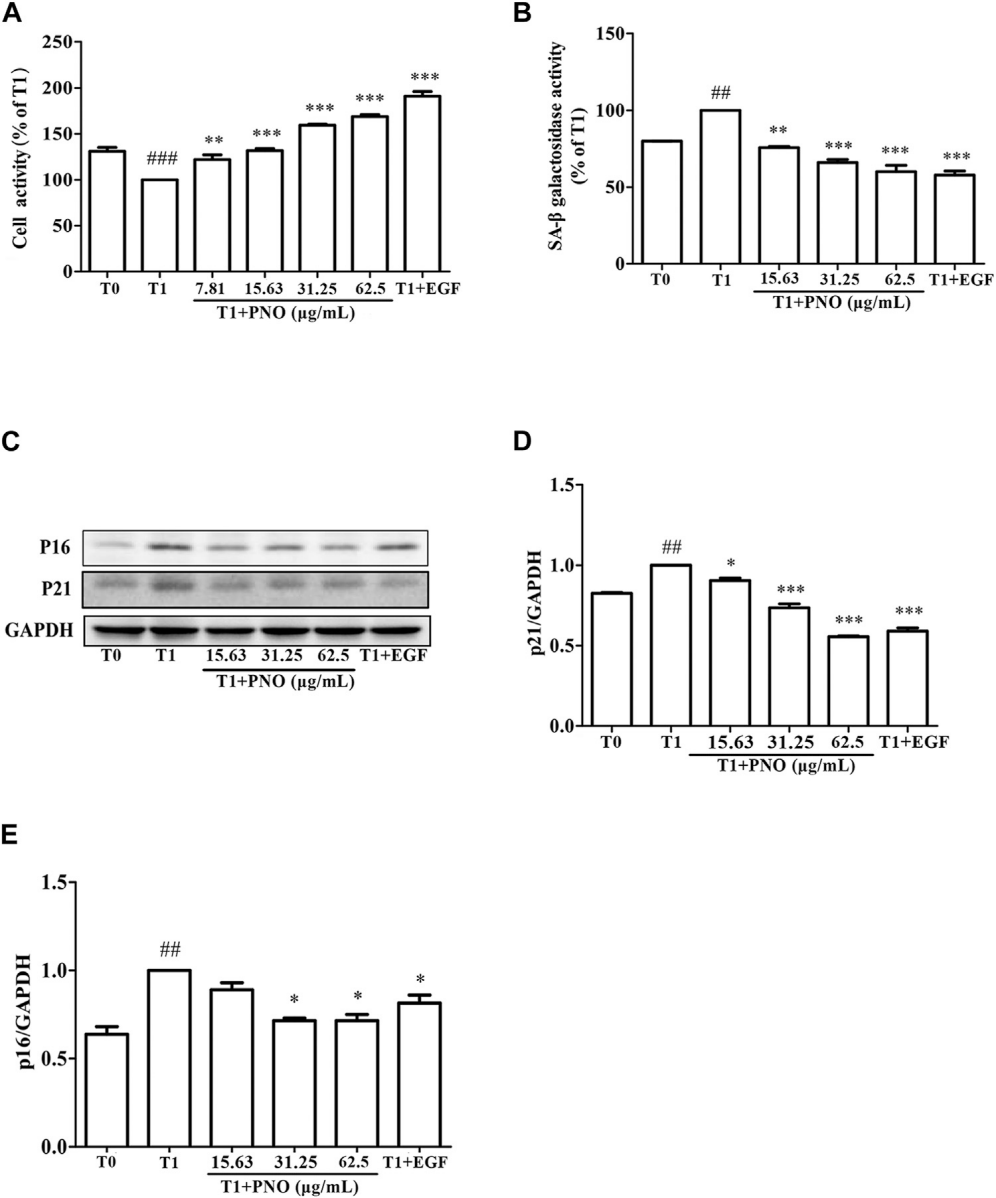
Effect of PNO on cell viability and replicative senescence of NIH-3T3 cells. **(A)** Effect of PNO on cell viability of replicative senescent NIH-3T3 cells. **(B)** Effect of PNO on SA-β-Gal activity of replicative senescent NIH-3T3 cells. **(C–E)** Effect of PNO on expression of p16, p21 proteins by replicative senescent NIH-3T3 cells as detected by western blotting (WB). Quantification of bands relative to GAPDH using Image J software. All data are presented as the mean ± SD of at least three independent experiments. ^#^ indicates a significant different relative to the T0 group. ^##^ *p* < 0.01, ^###^ *p* < 0.001. ^*^Indicates a significant different relative to the T1 group. ^*^ *p* < 0.05, **p < 0.01, ***p < 0.001.

As shown in Figure 3A, as compared with the T0 group, cell vitality of the T1 group was decreased by 30.9 ± 5.66%. In the T1+PNO group, PNO concentrations of 15.63, 31.25, and 62.5 μg/ml led to cell vitality increases of 24.92 ± 1.76%, 59.32 ± 1.93%, and 68.83 ± 3.14%, respectively, as compared with the T1 group. In the T1+EGF group, EGF increased cell vitality by 91.42 ± 7.34% relative to the T1 group. The results demonstrated that PNO could reverse reduced proliferative activity associated with dermal cell replicative senescence, indicating that PNO treatment could inhibit natural skin aging processes.

### PNO Inhibits Increases of SA-β-Gal Activity and Expression of p16 and p21 Proteins in Replicative Senescent NIH-3T3 Cells

As compared with the T0 group, secreted SA-β-Gal activity of the T1 group was significantly increased. After adding various concentrations of PNO (15.63, 31.25, 62.50 μg/ml), SA-β-Gal activity decreased by 24.22 ± 0.65%, 33.95 ± 1.92%, and 39.96 ± 4.11% in the T1+PNO group, respectively, as compared to the T1 group. Treatment with EGF (50 ng/ml) led to a decrease of SA-β-Gal activity by 42.19 ± 2.74% in the T1+EGF group (Figure 3B). Different concentrations of PNO significantly inhibited SA-β-Gal activity in replicative senescent NIH-3T3 cells. As shown in Figures 3C–E, as compared with the T0 group, expression levels of p16 and p21 proteins in the T1 group were significantly increased. However, after treatment with PNO, expression levels of p16 and p21 proteins in cells decreased in the T1+PNO group, with EGF exerting the same effect. Thus, PNO treatment appeared to dampen increases in SA-β-Gal activity and boost expression of p16 and p21 proteins in NIH-3T3 cells undergoing replicative senescence. Based on the abovementioned results, it is apparent that PNO not only inhibited proliferative activity of replicative senescent cells, but also inhibited cellular aging processes.

### PNO Inhibits Cell Cycle Arrest Associated with Replicative Senescence of NIH-3T3 Cells

**FIGURE 4:**
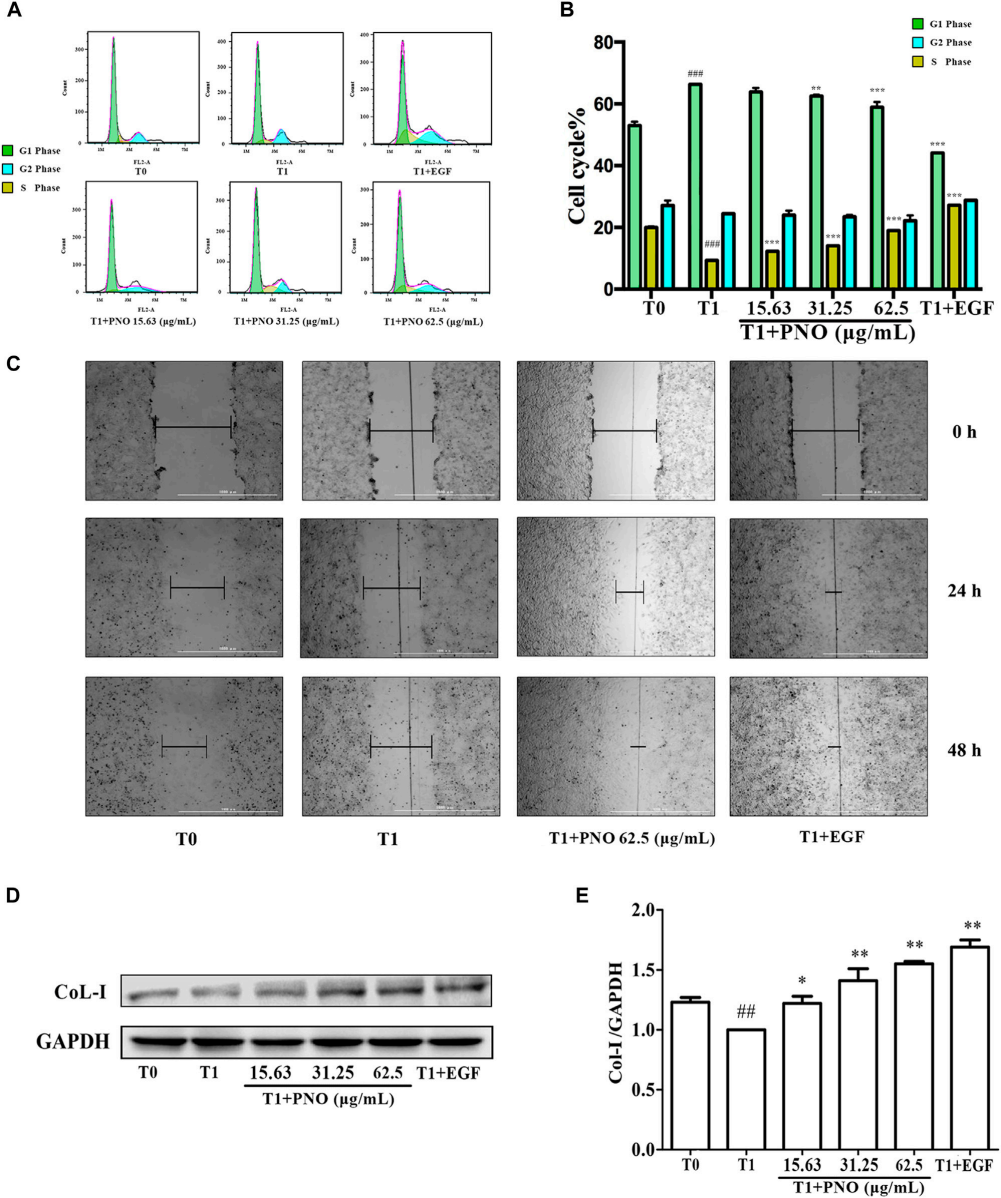
Effect of PNO on cell cycle progression and migration of replicative senescent NIH-3T3 cells. **(A**,**B)** Flow cytometry determined the effect of PNO on the cell cycle of replicating senescent NIH-3T3 cells. **(C)** The effect of PNO on migration of replication senescence NIH-3T3 cells. **(D**,**E)** Effect of PNO on expression of CoL-I proteins by replicative senescent NIH-3T3 cells as detected by western blotting (WB). ^#^ indicates a significant different relative to T0 group. ^##^ *p* < 0.01, ^###^ *p* < 0.001. * Indicates a significant different relative to T1 group. *p < 0.05, **p < 0.01, ***p < 0.001.

As shown in Figures 4A,B, as compared with the T0 group (G1 phase = 52.93 ± 2.41%, S phase = 19.95 ± 1.63%, G2 phase = 16.27 ± 0.39%), in the T1 group (G1 phase = 66.29 ± 2.10%, S phase = 9.27 ± 0.70%, G2 phase = 16.76 ± 0.13%) the number of cells in G1 phase increased while the number of cells in S phase significantly decreased. After various concentrations of PNO were added to cells, proportions of cells in S phase increased with increasing PNO concentration in the T1+PNO group (T1+ 15.63 μg/ml PNO group: G1 phase = 64.60 ± 3.65%, S phase = 12.22 ± 0.72%, G2 phase = 14.36 ± 1.06%; T1+ 31.25 μg/ml PNO group: G1 phase = 62.53 ± 0.66%, S phase = 14.01 ± 1.35%, G2 phase = 14.73 ± 2.65%; T1+ 62.5 μg/ml PNO group: G1 phase = 58.89 ± 2.91%, S phase = 18.96 ± 0.22%, G2 phase = 17.98 ± 1.90%), mirroring results obtained for EGF (G1 phase = 44.12 ± 0.37%, S phase = 27.14 ± 0.15%, G2 phase = 20.60 ± 0.79%). The results showed that PNO could increase the proportion of S phase cells and promote progression of cells from G1 phase to S phase, thus demonstrating that PNO could rescue cells in replicative senescence from cell cycle arrest.

### PNO Promotes Migration Ability of Replicative Senescent NIH-3T3 Cells

As shown in Figure 4C, the migration distance of the T1 group was lower than the T0 group. After treatment with 62.5 μg/ml PNO or EGF, migration distances significantly increased by 24 and 48 h in the T1+PNO and T1+EGF groups, indicating that PNO could obviously restore migration ability of NIH-3T3 cells in replicative senescence, as observed for EGF.

### PNO Increases Secretion of CoL-I in Replicative Senescent NIH-3T3 Cells

Compared with the T0 group, levels of CoL-I in the T1 group decreased. As compared with the T1 group, PNO in concentrations of 15.63, 31.25, and 62.5 μg/ml led to increased cellular expression of CoL-I in the T1+PNO group (Figures 4D,E) mirroring increased levels observed in the T1+EGF group. These results show that PNO could promote CoL-I production by NIH-3T3 cells undergoing replicative senescence as evidence that PNO treatment increased secretion of cell matrix components by senescent cells.

### PNO Promotes Expression of Cell Cycle-associated Proteins in Replicative Senescent NIH-3T3 Cells

**FIGURE 5:**
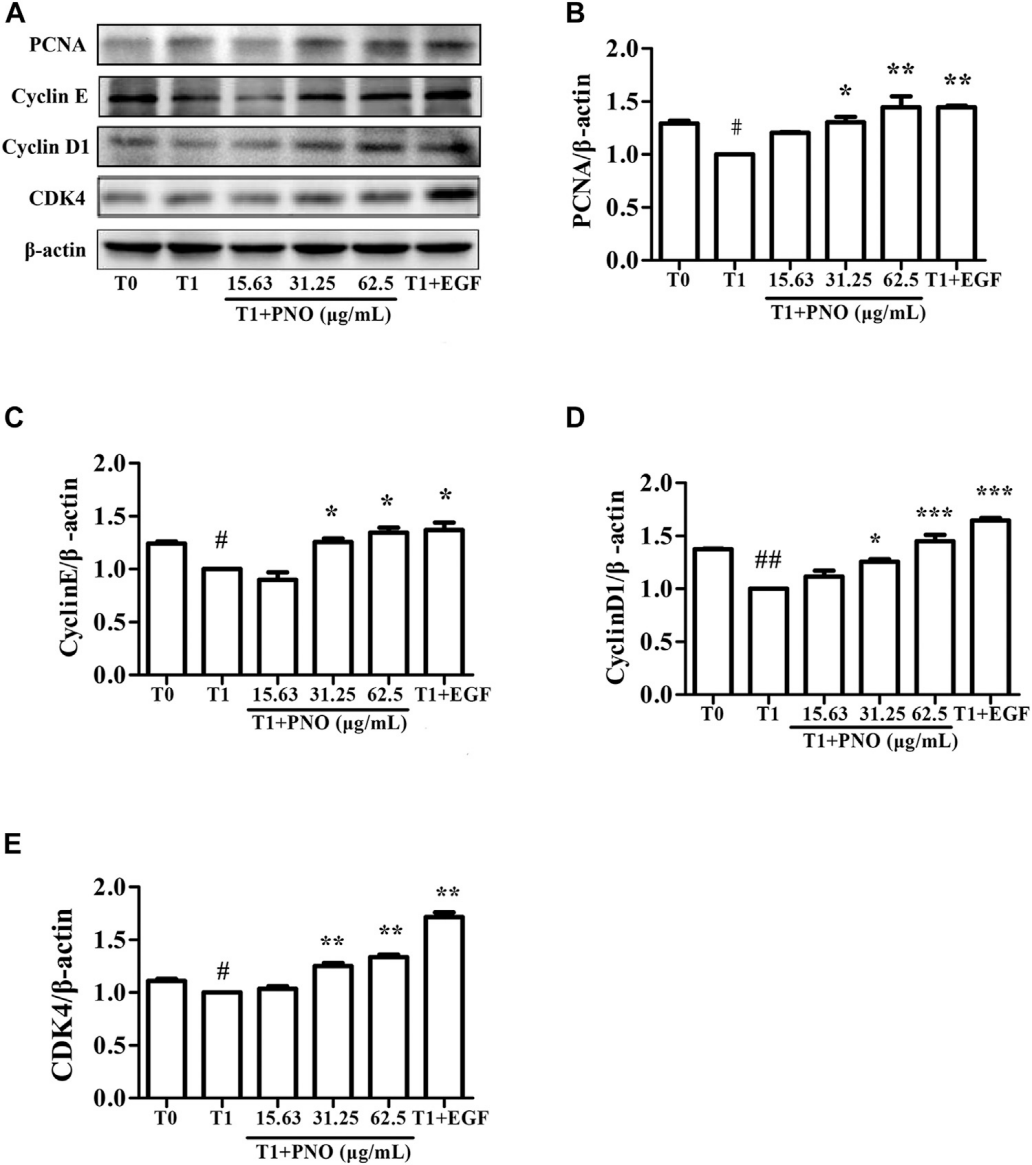
Effect of PNO on expression of cycle-related proteins in replicative senescent NIH-3T3 cells. **(A)** Western blotting analysis of expression of cycle-related proteins. **(B–E)** Quantification of bands relative to GAPDH using Image J software. All data are presented as the mean ± SD of at least three independent experiments. ^#^ indicates a significant different relative to T0 group. ^#^ *p* < 0.05, ^##^ *p* < 0.01, ^###^ *p* < 0.001. ^*^ Indicates a significant different relative to T1 group. ^*^ *p* < 0.05, **p < 0.01, ***p < 0.001.

As shown in Figures 5A–E, expression levels of PCNA, cyclin E, cyclin D1, and CDK4 in the T1 group were decreased as compared with corresponding levels in the T0 group. However, after treatment of cells with 31.25 and 62.5 μg/ml PNO, expression levels of all of these proteins significantly increased in the T1+PNO group. The results suggest that PNO treatment promoted cell cycle progression and accelerated cell proliferation by up-regulating expression of PCNA, cyclin E, cyclin D1, and CDK4, mirroring effects observed for the positive control (EGF-treated cells).

### PNO Activates Phosphorylation of Key Proteins in the MAPK Signaling Pathway in Replicative Senescent NIH-3T3 Cells

**FIGURE 6:**
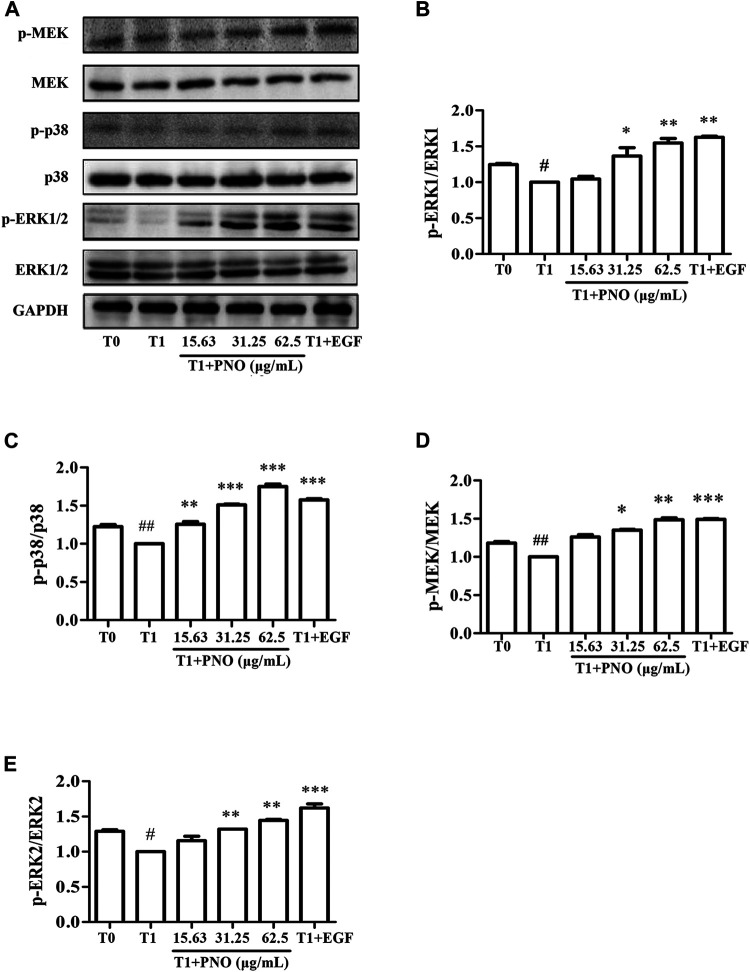
Effect of PNO on key proteins of MAPK signaling pathway in replicative senescent NIH-3T3 cells. **(A)** Western blotting analysis of expression of p-MEK/MEK, p-p38/p38, p-ERK1/2/ERK1/2. **(B–E)** Quantification of bands relative to GAPDH using Image J software. All data are presented as the mean ± SD of at least three independent experiments. ^#^ indicates a significant different relative to T0 group. ^#^ *p* < 0.05, ^##^ *p* < 0.01, ^###^ *p* < 0.001. ^*^ Indicates a significant different relative to T1 group. *p < 0.05, **p < 0.01, ***p < 0.001.

As compared with the T0 group, relative protein level ratios of p-MEK/MEK, p-p38/p38, and p-ERK1/2/ERK1/2 in the T1 group were reduced, while treatment of cells with 31.25 and 62.5 μg/ml PNO promoted phosphorylation of these proteins in the T1+PNO group (Figures 6A–E). Thus, PNO could promote phosphorylation cell cycle-related proteins in NIH-3T3 cells undergoing replicative senescence by activating a series of key protein kinases in the MAPK signaling pathway, mirroring the effect in the positive control (EGF-treated cells).

### PNO Promotes Phosphorylation of Migration Regulatory Proteins in Replicative Senescent NIH-3T3 Cells

**FIGURE 7:**
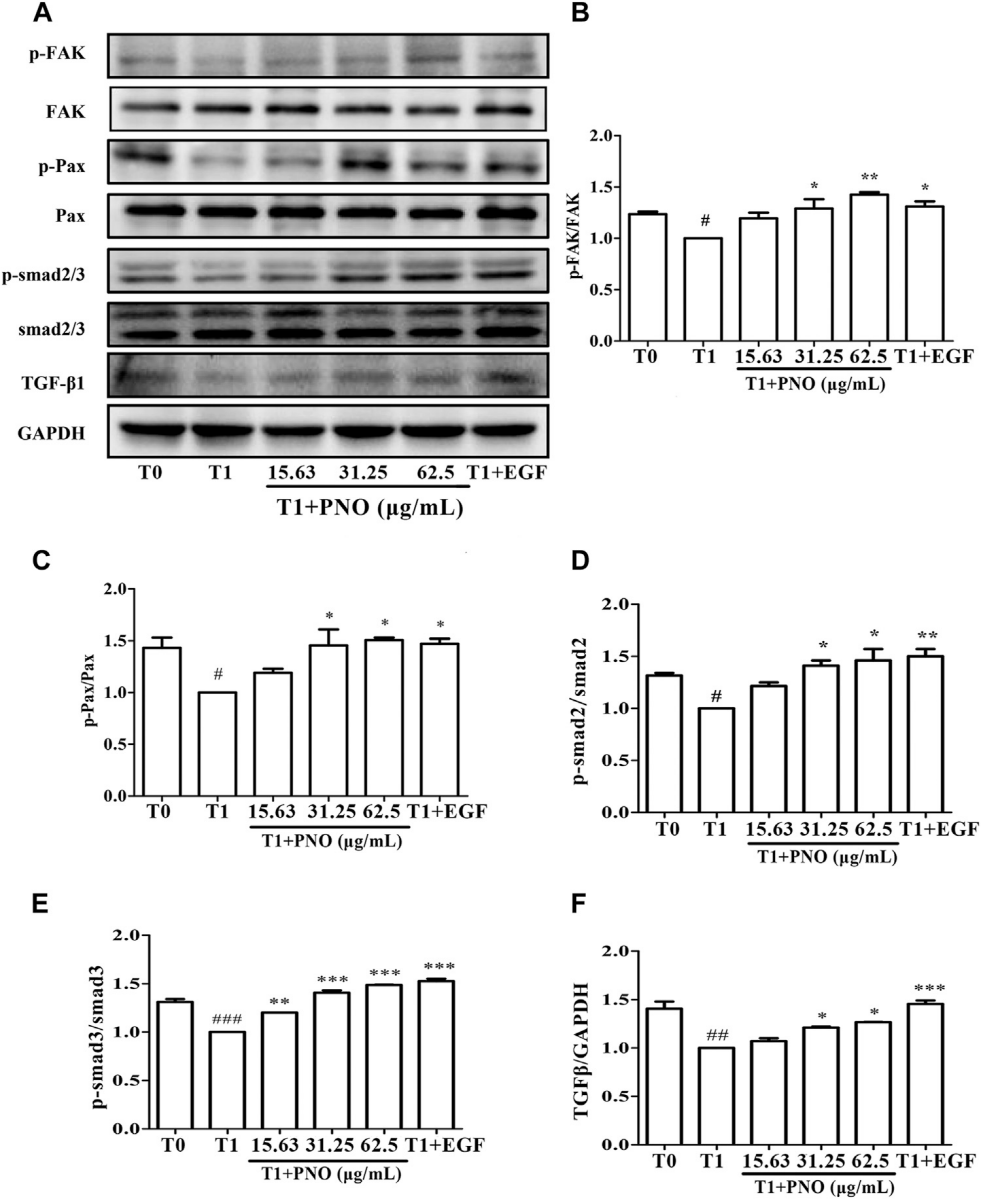
Effect of PNO on expression of migration regulatory proteins and TGF-β1/Smad signaling pathway in replicative senescent NIH-3T3 cells. **(A)** Western blotting analysis of expression of migration regulatory proteins and TGF-β1/Smad signaling pathway. **(B–F)** Quantification of bands relative to GAPDH using Image J software. All data are presented as the mean ± SD of at least three independent experiments. ^#^ indicates a significant different relative to T0 group. ^#^ *p* < 0.05, ^##^ *p* < 0.01, ^###^ *p* < 0.001. ^*^ Indicates a significant different relative to T1 group. *p < 0.05, **p < 0.01, ***p < 0.001.

As compared with the T0 group, p-FAK and p-Pax protein levels were significantly reduced in the T1 group. After treatment of the T1+PNO group with 31.25 and 62.5 μg/ml PNO, increases in expression levels of p-FAK and p-Pax proteins were observed as compared to corresponding T1 group levels (Figures 7A–C), mirroring the effect observed for the positive control (EGF-treated cells). This result indicates that PNO could promote phosphorylation of FAK and Pax proteins in NIH-3T3 cells undergoing replicative senescence, leading to increased cell migratory activity.

### PNO Activates the TGF-β1/Smad Signaling Pathway in Replicative Senescent NIH-3T3 Cells

Compared with the T1 group, TGF-β1, p-Smad2, and p-Smad3 protein levels were significantly increased in cells treated with 31.25 and 62.5 μg/ml PNO, mirroring the effect observed for EGF-treated cells in the T1+PNO group (Figures 7A,D–F). Thus, PNO appeared to activate the TGF-β1/Smad pathway, leading to increased phosphorylation of FAK and Pax proteins and subsequently increased synthesis of CoL-I in replicative senescent NIH-3T3 cells.

## Discussion

In this study, by exploiting properties of chemical polarity and molecular weight, we isolated oligosaccharides from *P. notoginseng* in a stepwise manner using ethanol precipitation, anion exchange chromatography, HPLC, and gel column chromatography. Ultimately, PNO treatment boosted cell proliferation activity, migration, and CoL-I synthesis in undergoing replicative senescence. These results suggest that this active preparation exerted an effective skin anti-aging effect that has not been reported previously for oligosaccharides. However, recently it has been reported that oligosaccharides from various sources exhibited anti-photoaging healing effects when used to treat dermal wounds (Bouaziz et al., 2014; Kong et al., 2018; Suh et al., 2020). For example, chitosan oligosaccharide attenuated UV-induced skin photoaging by promoting favorable regulation of antioxidant and anti-inflammatory activities (Kong et al., 2018). Meanwhile, treatment with oligosaccharides generated from almond gum (*Prunus amygdalus*) applied alone or as a cream-based formulation have been shown to accelerate wound healing (Bouaziz et al., 2014). Here we found PNO to be mainly composed of mannose and galactose, with a small proportion of sorbitose; this finding aligns with results of other studies showing mannose and galactose contents to be high in many oligosaccharides, but with relative monosaccharide proportions and compositions varying among oligosaccharide preparations (Yang C. et al., 2020; Yu et al., 2021). Notably, mannose, a monosaccharide widely distributed within many human and plant tissues, can inhibit wound inflammation and accelerate wound healing in diabetic mice (Wei et al., 2020). Meanwhile, galactose has been commonly used to induce senescence *in vitro* and *in vivo* (Azman and Zakaria, 2019). Intriguingly, sorbitose has not been reported as a component of other oligosaccharides, but has been reported to decrease lipid metabolism in rats (Nagata et al., 2018). Thus, sorbitose may account for the characteristic activity of PNO. Other *P. notoginseng* components have been reported to exert anti-aging effects when used to treat many degenerative diseases associated with aging. For example, notoginsenoside Rg1 and R1 improved cognitive impairment and increased neuronal excitability against AD neurotoxicity (Yan et al., 2014; Liu et al., 2019), while other notoginsenosides attenuated cardiomyocyte apoptosis via the mitochondrial pathway in naturally aging rats (Zhou et al., 2018). In addition, notoginseng extracts delayed vascular endothelial cell senescence (Wang and Lei, 2014), while active polysaccharide ingredients of *P. notoginseng* significantly extended the lifespan of *C. elegans* (Feng et al., 2015). Notably, in one anti-aging skin treatment study, administration of *P. notoginseng* (referred to as ginsenosides extracted from Sanchi) significantly diminished facial wrinkles and alleviated other symptoms of facial skin aging (Rattan et al., 2013). However, notoginseng active ingredients and their effects on skin fibroblast replicative senescence have not yet been reported, prompting this study. In this work, the effect of *P. notoginseng* oligosaccharides on replicative senescence was investigated using a skin aging model based on replicative senescent NIH-3T3 fibroblasts. The methodology used here to study aging will likely be of great significance for enhancing our understanding of anti-aging substances, including *P. notoginseng* oligosaccharides by serving as a systematic framework for evaluating efficacy and exploring mechanisms of action.

During skin aging, fibroblast replicative senescence occurs, which has been defined as a cellular state of stable cell cycle arrest with long-term loss of proliferative capacity (Yuan et al., 2020). Levels of a biomarker associated with cell senescence, SA-β-gal activity, have been shown to be increased in cells in a replicative senescent state. Increased SA-β-gal activity results from an increase in lysosome number and size that consequently leads to elevated senescent cell lysosome content (Mohamad Kamal et al., 2020). During extreme senescence, gene expression profiles of cell cycle inhibitors and cyclin-dependent kinase inhibitors (CDKIs) p21 and p16 are altered at the transcriptional level, resulting in lower production of cell cycle stimulatory proteins and other markers associated with cellular senescence. Here we found that PNO treatment of replicative senescent NIH-3T3 cells could promote cell vitality and relieve cell cycle arrest, while also inhibiting SA-β-gal activity and expression of p21 and p16 proteins. Taken together, these *in vitro* results suggest that PNO may promote proliferation of cells undergoing replicative senescence to reverse cellular aging, with relevance to alleviation of natural skin aging processes *in vivo*.

Notably, human fibroblast G1 phase cell cycle arrest occurs prior to cell entry into the senescent state, which results from increases of senescence-related proteins such as p16 and p21 that inhibit binding of CDK to cyclin D1 and cyclin E to subsequently prevent G1/S cell cycle progression (Chen et al., 2018; Sadowska-Bartosz and Bartosz, 2020). Several studies have demonstrated that production of cyclins is a major downstream consequence of MAPK signal pathway activation (Wang C. et al., 2020). Meanwhile, activation of MEK triggers downstream phosphorylation of ERK and p38, which, in turn, promote synthesis of PCNA protein that then induces cell proliferation (Li et al., 2020). Intriguingly, experimental results obtained here demonstrated that PNO treatment could indirectly promote expression of CDK4, cyclin D1, cyclin E, and PCNA proteins in replicative senescent NIH-3T3 cells *via* activation of the MAPK signaling pathway.

Another indicator of replicative senescence, decreased cell migratory activity, is a process that is mainly regulated by local focal adhesion kinase (FAK) and paxillin (Pax) protein activities. In non-senescent cells, Pax activation by p-FAK promotes cell migration. (Cerny et al., 2018). Activation of FAK/Pax is mainly regulated by the TGF-β1/Smad signaling pathway (Cheng et al., 2020); this pathway is activated when TGF-β1 binds to the TGFR receptor to induce Smad2/3 phosphorylation which, in turn, stimulates production of nuclear transcription factors that stimulate integrins production and activate FAK and Pax to promote cell migration (Bukowska et al., 2018). Notably, the TGF-β1/Smad signaling pathway also regulates production of CoL-I. Here we demonstrated that CoL-I secretion by replicative senescent NIH-3T3 cells could occur after cells are activated *via* the TGF-β1/Smad signaling pathway; thus, activation of TGF-β1/Smad signaling may delay cell entry into replicative senescence. In addition to stimulation TGF-β1/Smad signaling and CoL-I secretion, here we demonstrated that PNO treatment could promote cell migration and increase generation of p-FAK and p-Pax. Therefore, the abovementioned results suggest that PNO promotes cell migration and CoL-I production by activating the TGF-β1/Smad signaling pathway as the mechanism whereby PNO alleviates replicative senescence.

## Conclusion

An oligosaccharide mixture that delayed replicative senescence of NIH-3T3 fibroblasts was prepared from *P. notoginseng* (PNO). PNO was shown to activate the MAPK pathway to induce synthesis of cell cycle regulatory proteins that promoted cell proliferation, cell migration and production of CoL-I. PNO was shown to alleviate replicative senescence by activating the TGF-β1/Smad signaling pathway as an underlying mechanism exploited by PNO to delay cell entry into a replicative senescence state and slow skin cellular aging-related processes (Supplementary Figure 1).

## Data Availability

The original contributions presented in the study are included in the article/Supplementary Material, further inquiries can be directed to the corresponding authors.

## Abbreviations

PNO, Panax notoginseng oligosaccharides; PCNA, cell proliferation antigen; CDK4, cycle-dependent kinases 4; CoL-I, type I collagen; TGF-β1, transforming growth factor-β1; FAK, focal-adhesion kinase; Pax, paxillin; ECM, dermal extracellular matrix.
