# Supplementary figures and images for: A Novel Biochemical Study of Anti-Dermal Fibroblast Replicative Senescence Potential of Panax Notoginseng Oligosaccharides
Source: Front Pharmacol. 2021 Jun 30;12:690538. doi: 10.3389/fphar.2021.690538 (PMC8277921; doi:10.3389/fphar.2021.690538)

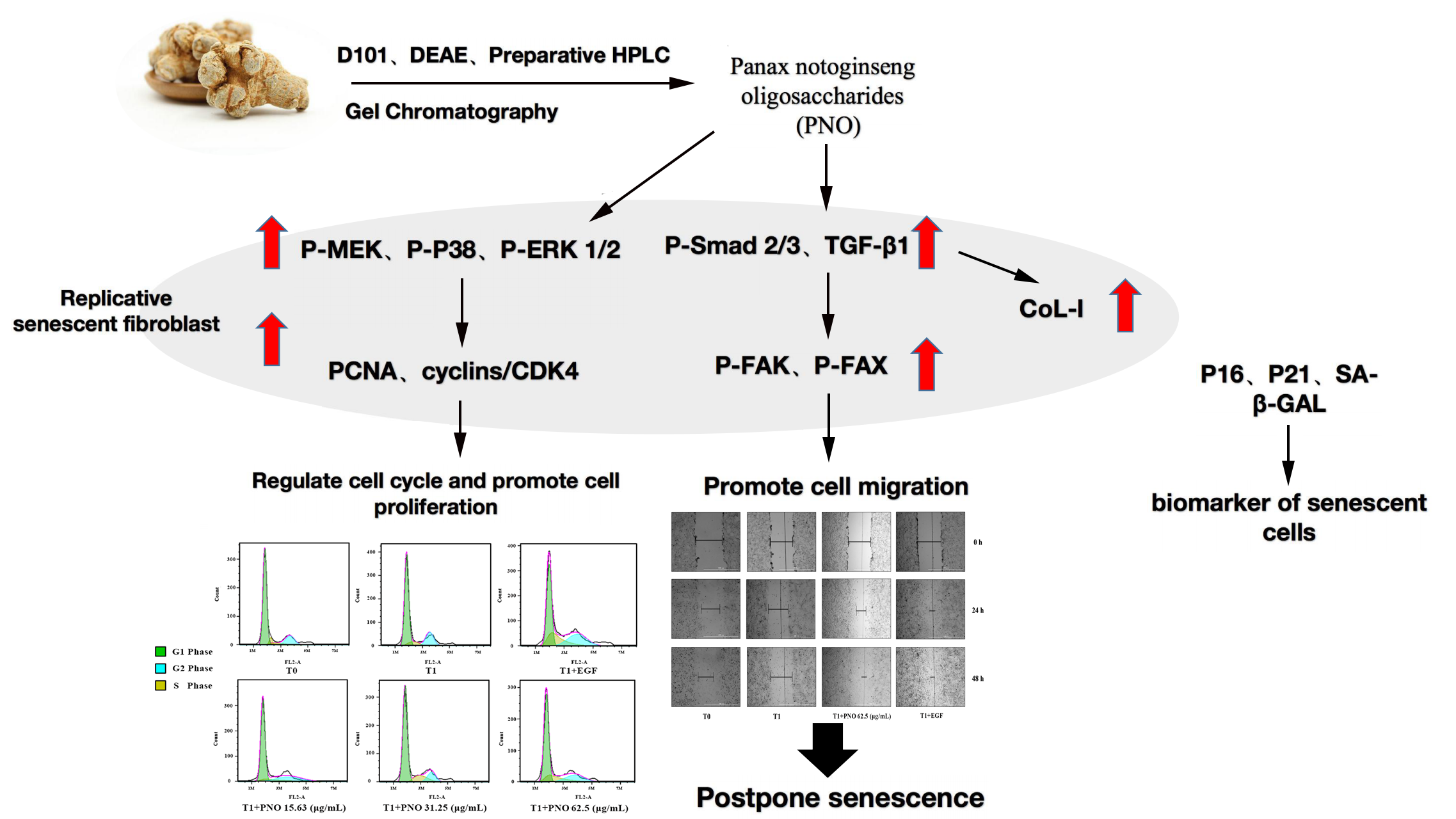

Supplement: Supplementary file 1 [file Image1.tif]
